# Deep-learning based representation and recognition for genome variants—from SNVs to structural variants

**DOI:** 10.1093/nsr/nwae335

**Published:** 2024-09-19

**Authors:** Songbo Wang, Kai Ye

**Affiliations:** School of Automation Science and Engineering, Faculty of Electronic and Information Engineering, Xi'an Jiaotong University, China; MOE Key Lab for Intelligent Networks & Networks Security, Faculty of Electronic and Information Engineering, Xi'an Jiaotong University, China; School of Automation Science and Engineering, Faculty of Electronic and Information Engineering, Xi'an Jiaotong University, China; MOE Key Lab for Intelligent Networks & Networks Security, Faculty of Electronic and Information Engineering, Xi'an Jiaotong University, China; School of Life Science and Technology, Xi'an Jiaotong University, China; Faculty of Science, Leiden University, The Netherlands; Genome Institute, The First Affiliated Hospital of Xi'an Jiaotong University, China

The evolution of genome sequencing and artificial intelligence (AI) has ushered in a new era of variant calling. Deep-learning methods have notably advanced the detection of both small-scale and large-scale variants, overcoming limitations and unresolved issues faced by traditional methods built on statistics and modeling. Here, we review eight deep-learning variant callers, deconstructing their computational procedures into two key modules, representation and recognition. The representation module takes genome sequencing data as input and encodes genomic features with greater depth. The recognition module, built upon deep-learning models, tackles complex variant detection tasks and outputs variant properties with superior accuracy. This perspective aims to pave the way for leveraging AI in future variant research.

In genetics, genome variants hold the key to understanding the differences in DNA makeup among individuals. These variations fall into two main categories [1]: (1) small-scale variants, typically <50 base-pairs (bps), include single-nucleotide variants (SNVs) and short insertions and deletions (Indels). (2) Large-scale variants encompass structural variants (SVs) and complex SVs (CSVs) >50 bps. Both categories can significantly impact biological processes, potentially leading to diseases or diverse phenotypes [1].

The rise of genome sequencing has spurred the development of numerous variant callers [2]. Traditional methods, inspired by reductionism, establish simplified models patched with redundant constraint rules. While they have unearthed a wealth of variants and implications, these incomplete and biased rules are difficult to fully address real-world scenarios, leading to omissions, errors and a lack of generalization among applications. However, the application of AI, particularly deep-learning methods, has revolutionized specific aspects of variant calling. Based on holism, these methods leverage high-level and integrated genome features for unbiased variant calling, particularly efficient for resolving complex variants that disrupt gene structures and complicate genome regions often discarded previously. Benefiting from significant advances in methodology, deep-learning callers demonstrate notable performance improvement, retrieving the missed but key variant sites while meanwhile correcting the artificially reported variant properties. Moreover, they provide insights into novel variant types and their crucial effects on the genome, enhancing understanding of the relationships between variants and diverse phenotypes or diseases.

This perspective surveys ten recently published deep-learning callers (Fig. 1a), including five small-scale variant callers (DeepVariant [3], NeuSomatic [4], PEPPER [5], DeepMosaic [6], Clair [7], Clair3 [8] and NanoCaller [9]) and three large-scale variant callers (SVision [10], Cue [11] and SVision-pro [12]). We aim to provide a comprehensive overview of their functionalities and how they differ from traditional callers. This knowledge will be instrumental in maximizing the potential of AI in future variant research.

**Figure 1.**
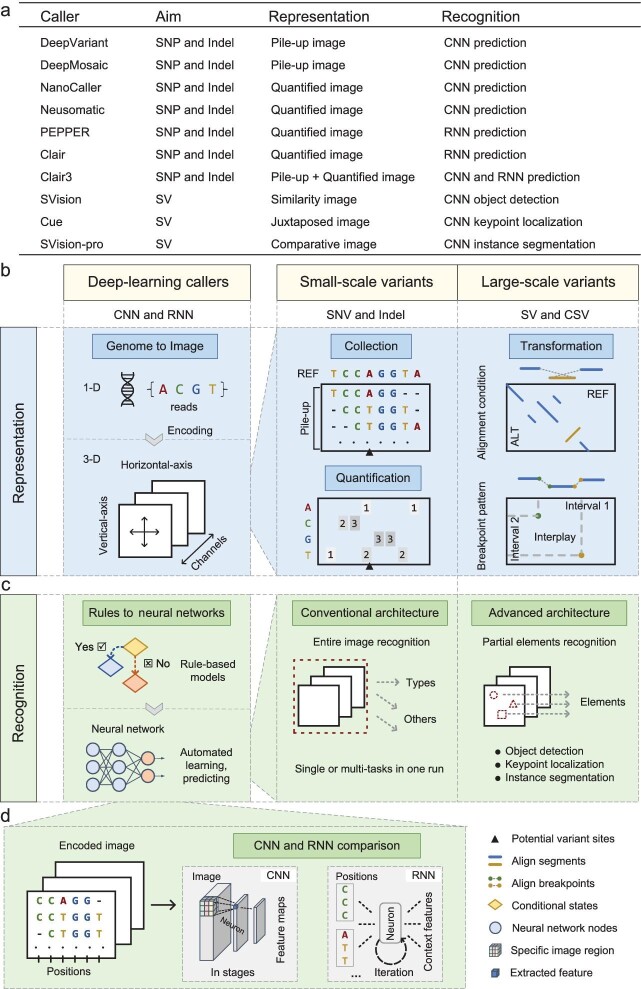
Illustration of deep-learning based representation and recognition for genome variant calling. (a) Collection of reviewed callers; (b) the representation module; (c) the recognition module; (d) the comparison between CNN and RNN in process encoded images.

While traditional callers for small-scale and large-scale variants exhibit wide divergence, deep-learning callers generally share two key modules (Fig. 1b and c): (1) representation, encoding the raw genome sequencing data into feature-rich images, and (2) recognition, employing neural networks to analyze these encoded images and identify variant properties, such as variant type and genotype.

## REPRESENTATION MODULE: ENCODING GENOMES INTO IMAGES

Genome sequencing produces vast amounts of DNA reads composed of just four bases. However, due to varied read lengths and the presence of sequencing errors, instrument biases and alignment artifacts, these reads are unsuitable for direct input into deep-learning methods. Therefore, deep-learning callers first encode genomes into images by collecting, quantifying and transforming features from sequencing reads (Fig. 1b).

**Collecting and quantifying for small-scale variant representation**. Small-scale variants (SNVs and Indels) indicate localized alterations in DNA base-pairs. Therefore, deep-learning callers for these variants focus on collecting and quantifying features around potential variant sites. These features are then encoded into images, where the horizontal-axis represents genome locations and the vertical-axis represents the features.

- Pile-up images: DeepVariant and DeepMosaic use pile-up images. The reference genome and sample reads are collected and then displayed vertically by assigning each base type with a unique pixel color. Additional features, such as base quality scores and mapping quality scores, are encoded along separate channels. This approach resembles how genomic researchers visually curate variants using Integrated Genome Views (IGVs), promoting ease of understanding and usability.
- Quantified images: other callers (Clair, NanoCaller, NeuSomatic and PEPPER) additionally quantify base types and their frequencies after pile-up. Consequently, the vertical-axis typically counts of each base type for each genome position along the horizontal-axis. Additional features could be either appended along the vertical-axis (e.g. PEPPER), added in channels (e.g. NeuSomatic and NanoCaller), or both (e.g. Clair). While less intuitive, this method allows for smaller image sizes, reducing both computational and storage costs. Furthermore, quantified images allow for capturing long-range haplotype information, as demonstrated by NanoCaller, which counts solely heterozygous positions spanned by the same reads. This highlights the advantage of long reads over short reads, which only provide information on neighboring bases.

The two types of images can be integrated, as done by Clair3, where lightweight quantified images fast-screen the entire genome, and detailed pile-up images subsequently retrieve low-quality candidates.

**Transforming for large-scale variants representation.** Large-scale variants (SVs and CSVs) involve extensive rearrangements of genomic segments (>50 bps). Representing these variants requires sophisticated strategies to transform alignment conditions or breakpoint patterns within reads.

- Similarity images: SVision visualizes the alignment conditions in similarity images, where lines and gaps represent aligned and unaligned segments between the variant sequence (VAR, at vertical-axis) and the reference sequence (REF, at horizontal-axis). The duplicated- and inverted-alignments are displayed in two separate channels. Additionally, SVision incorporates image de-noising by subtracting the VAR-to-REF image from the REF-to-REF image, to avoid redundant alignments caused by repetitive sequences. The similarity images mimic how experts manually inspect variants with dot-plots.
- Juxtaposed image: Cue uses two genome intervals as the horizontal and vertical axes of one image, respectively. Each image pixel corresponds to the features extracted from respective positions of the two intervals. The first channel's pixel values depict the read depth difference between the two locations. The remaining channels highlight how the two locations are connected by the breakpoints of discordant alignments. By focusing on breakpoint patterns instead of overall alignment conditions, Cue offers flexibility across different sequencing platforms (short-, long- and linked-read sequencing).
- Comparative image: SVision-pro integrates visualization and inter-sample comparison within one encoded image. It expands the similarity image concept by filling sample-specific genome information into empty image regions. Sample-specific information is represented as augmented-coverage-tracks (ACTs), where the raw IGV coverage tracks are augmented in colors according to the forward-, inverted- and duplicated-alignment conditions of reads. The filling regions are determined as two fixed-height empty tracks above and below the structures of similarity images, respectively. Therefore, each genome position in the image represents both the SV structure and its inter-sample differences.

## ADVANTAGES OF IMAGE REPRESENTATION

- **High dimensional feature augmentation**. Compared to the one-dimensional reads used by traditional callers, images allow for stacking multiple features at variant regions, leading to higher detection accuracies and reduces false-positives caused by sequencing errors. This is particularly beneficial for large-scale and complex variants, which are difficult to be described in one-dimensional reads but can be effectively illustrated in images with more dimensions.
- **Flexible extendibility**. Image representation offers flexibility for diverse scenarios involving different sequencing technologies and sample numbers. For example, traditional callers often focus on a single sequencing technology, while Cue can handle data from three different technologies by encoding breakpoint patterns. Additionally, an image can be easily adapted for multiple-sample calling by concatenating multiple images along either horizontal-, vertical- or channel-axis, enabling various types of applications.

## RECOGNITION MODULE: IDENTIFYING VARIANT PROPERTIES

Following the representation stage, a common approach is to pass the encoded images through feature extractors and then classifiers or regressors, and treat them as distinct layers within a neural network (Fig. 1c). Two architectures are broadly employed: convolutional neural networks (CNNs) and recurrent neural networks (RNNs). CNNs draw inspiration from animal visual cortices. Each convolutional neuron independently responds to a specific image region and extracts features. In contrast, RNNs leverage a recurrent feedback mechanism to learn relations from time-series-like data, capturing genome context rather than focusing solely on individual image regions (Fig. 1d). In terms of the accomplishable tasks, conventional CNNs and RNNs predict the entire encoded images into one or several predefined classes, while advanced neural networks recognize partial elements within the encoded images rather than the entire image at once, therefore addressing more complicated tasks.

**Conventional neural networks for small-scale variants**. The types of small-variants, such as SNVs and Indels are well explored. And therefore, conventional neural networks are adequate for identifying them.

- Conventional CNNs implement multiple convolutional layers to extract image features in stages and then feed the feature maps into fully-connected-layer based classifiers for identifying variant properties. For instance, DeepVariant utilizes the CNN for variant genotype classification task, and DeepMosaic for mosaic variants detection task.
- Conventional RNNs recurrently iterate each genome position in the encoded images to extract context features. Clair flattens context features of all genome positions in the image and then, like conventional CNNs, inputs them into classifiers. PEPPER exploits the major characteristic of RNNs, outputting the most likely base type for each genome position during the iteration. Therefore, PEPPER corrects potential mis-callings caused by sequencing errors and works well on error-prone sequencing data.

These conventional CNNs and RNNs can also accomplish regression tasks (e.g. predicting variant lengths or locations) by using regressors that yield continuous values rather than discrete classifications. They are also capable of recognizing multiple variant properties in parallel when combining multiple classifier and regressor layers. For example, NeuSomatic employs two classifiers and one regressor, and Clair employs four classifiers.

**Advanced neural networks for large-scale variants**. Large-scale variants (SVs and CSVs) encompass multiple breakpoints or sub-components, making their identification challenging. Studies use advanced neural networks to address this.

- SVision introduces the object detection methodology into CSV detection problem. Assuming that CSVs consist of five basic SV sub-components, SVision first splits the similarity image into sub-images. A conventional CNN then classify each sub-image into its corresponding component type. All recognized sub-components are finally combined for the identification of CSV.
- Cue formulates the SV discovery problem as a multi-class keypoint localization task, where keypoints correspond to SV breakpoints in encoded images. It utilizes a stacked hourglass CNN to extract features at multiple resolutions, thereby capturing both individual breakpoints and interplay patterns indicative of CSVs. Cue outputs a set of confidence maps, classifying breakpoints into SV types and genotypes.
- SVision-pro leverages image instance segmentation for one-stop type detection and 3-way comparison between samples. Its encoder-decoder CNN classifies each image pixel into one of the five basic SV component classes or a background class. The inter-sample comparison is achieved by comparing the regions of same-class pixels, where the horizontal span represents SV length, while the vertical span represents its allele fraction.

## THE ADVANTAGES OF RECOGNITION

**Automating detection**. Traditional callers, like decision trees, rely on manual rules for variant identification. Neural networks replace this subjective process with automated parameter learning and adjustment, which also enable easy switches between sequencing technologies (e.g. error-prone and high-fidelity long-reads) through using multiple trained models, rather than requiring specifically designed modules. Additionally, the interpretability of neural networks, as explored in DeepMosaic and SVision-pro, facilitates the backtracking of decision-making processes, leading to a better understanding of detection differences among variants.

**Tackling complex tasks**. Equipped with multiple classifiers or regressors, neural networks can address multiple tasks simultaneously, outputting comprehensive variant properties in one run. These advanced neural networks can even address novel variant types, facilitating the discoveries of new gene-relevant events. For instance, SVision identified three distinct alleles (wild type, SV and CSV) at the neural-development gene *CNTN5* and the CSV allele caused a duplicated exon signature in its transcript.

This perspective examined eight deep-learning variant callers and summarized two core modules that differentiate them from traditional methods: representation and recognition. In brief, representation takes advantage of images for comprehensive genome feature acquisition, and recognition leverages powerful deep-learning for automated and accurate variant detection. These modules overcome the limitations of manual rule-based traditional approaches. Additionally, the interpretability of neural-networks can aid researchers in understanding the decision-making process behind variant identification. Deep-learning has demonstrably advanced variant calling while it can continue to propel variant calling and contribute to human health if population- and disease-relevant variant discovery could be prioritized. Population variant analysis often struggles with high false-positives due to the callset-level merging. Using images to represent the genomic differences among multiple samples and implementing deep learning genotyping models are essential to address this challenge. Disease-relevant research often requires the detection of low-allele variants whose sequence signals are indistinguishable from background noises due to sequencing errors and repetitive regions. Therefore, future research may use image representation for signal denoising and capturing accomplished by deep learning recognition to promote variant discovery in clinical applications.
